# The primary nursing implementation in late preterm

**DOI:** 10.1186/1824-7288-40-S2-A48

**Published:** 2014-10-09

**Authors:** R Ongaretto, F Villardino, F Faggion, S Tosatti, A Croso

**Affiliations:** 1 Nurse Neonatal Intensive Care Unit ASLBI Hospital Biella, Italy; 2 Chief Nurse Neonatal Intensive Care Unit ASLBI Hospital Biella, Italy; 3 Nursing Care Director ASLBI Hospital Biella, Italy

## Background

The Primary Nursing is a nursing care policy based upon an individualized patient-nurse relationship, with a particular attention to the communication and the continuity of the health care. This model has been implemented in all the structures of the ASL of Biella in 2013. The nurse becomes the coordinator of the patient’s assistance. The Primary Nursing objective is to develop the principles of self-care and enpowerment through taking care of the person and the continuity of care. The patient care is committed to a specific nurse (primary nurse) who is responsible for him during the hospitalisation.

The primary nurse, planning a personalized and continuative assistance in cooperation with the patient, the family and the health staff, is the point of reference, not only for the patient and the family, but also for the health team.

## Objective

The introduction of the Primary Nursing policy in the Neonatal Intensive Care Unit aimed at:

1) increasing the satisfaction:

- of patients and families who are better informed and supported by an individualized and qualified assistance

- of nurses because the increase responsibility raises their competence and the ability to cooperate in the health staff

2) developing the continuity of the health care by increasing the parent’s self government in the late preterm care and the quality of nursing management

## Material and methods

The method chosen for the care policy trial can be divided in two parts:

the general area ; the paediatric nurses take part to the different steps of the hospital health care strategy defined by the guidelines of the literature (FAD residential practical training)

the specific area with the achievement of nursing plans, tools and monitoring systems for the application and the evaluation of the policy in the specific area

## Results

We analysed 79 medical records, that is all the newborns discharged from 1^st^ September 2013 to 31^st^ March 2014. Outcomes highlight that 64.6% of the patients have been taken in care by a PN ; that 39.2% of prescriptions have been planned; the 53% of the latters has been followed by the associated nurses. Conflicts between nurses have been observed in only 3.9% of the cases.

## Conclusions

in the analysis of data , we notice a constant improvement of outcomes in the nursing care plan compiling. We intend to propose a new period of data collection in 2015.

